# From administrative data to action: Why Ecuador needs an economic burden assessment on child maltreatment

**DOI:** 10.3389/fpubh.2026.1880886

**Published:** 2026-10-07

**Authors:** Fiorella Quiroz-Cárdenas, Doris Quiroz-Cárdenas, José Francisco López-Gil

**Affiliations:** 1 Vicerrectoría de Investigación y Postgrado, Universidad de Los Lagos, Osorno, Chile; 2 School of Medicine, Universidad Espíritu Santo, Samborondón, Ecuador; 3 Centro de Salud Chimbacalle, Ministerio de Salud Pública, Quito, Ecuador

**Keywords:** Ecuador, child maltreatment, economic burden, child protection services, public health

## Abstract

Child maltreatment is a major public health problem with lasting health and socioeconomic consequences, yet economic-burden evidence from Latin America remains scarce. Using March 2026 administrative records from Ecuador's Ministry of Human Development, we annualized projected governmental expenditure across three Special Protection Services modalities, institutional care, foster care, and family support, serving 3,588 registered children. The base projection was approximately US dollars (USD) 25.4 million per year (about USD 7,087 per registered user). This figure captures institutional programme expenditure only and is not a national economic-burden estimate of child maltreatment, because services address multiple protection needs and exclude health, education, justice, family, and productivity costs, as well as children outside the formal system. We argue that linked administrative data across protection, health, and education sectors, together with nationally representative prevalence measurement, are required to estimate the economic burden of child maltreatment in Ecuador and to inform evidence-based policy.

## 1 Introduction

Child maltreatment represents a major public health concern with far-reaching consequences for individuals, families, and societies. It refers to the abuse and neglect experienced by individuals under 18 years old, which leads to actual or potential harm to children's health (1). Literature suggests that children who have experienced life stressors and traumatic events have sleep and mood disturbances (2), and even an increased risk of premature mortality (3). Similarly, this population is likely to experience significant long-term economic disadvantages, including elevated risks of unemployment, restricted access to skilled or professional employment, reduced lifetime earnings, and diminished asset accumulation (4).

Child maltreatment has major health effects and imposes an economic burden that goes far beyond government expenditure for child protection systems. Within a health economics framework, government expenditure refers to public resources allocated to provide goods or services associated with a particular condition, and it can be estimated from service utilization and the corresponding cost per unit of service (5). In contrast, the economic burden refers to costs and the economic consequences related to a condition or disease on individuals, families, governments, and society, while cost-of-illness (COI) studies provide a methodical framework for estimating that burden (6). COI generally incorporates relevant costs such as direct medical costs, direct non-medical costs, and, when possible, indirect costs related to disability, productivity losses, or premature mortality (3, 6). Such estimates provide important evidence for informing decision-making and resource allocation in public policy (6, 7). In contrast, cost-effectiveness analyses are a type of economic valuation that compares the costs and health outcomes of alternative interventions (7). Therefore, it addresses a different analytical question from estimating economic burden. Budget impact analysis, which estimates the net financial consequences of adopting an intervention within a defined budget and time horizon, is beyond the scope of this Perspective (8).

Recent systematic reviews have demonstrated that economic evaluations of child maltreatment have focused mainly on preventive and cost-effectiveness interventions in high-income countries (9–12), whereas studies quantifying its economic burden remain scarce. Peterson et al. (3) estimated that the economic burden of child maltreatment in the United States was approximately US dollars (USD) 831,000 per child, with a total social cost close to USD 2 trillion, consistent with earlier United States lifetime estimates (13). International lifetime societal cost estimates are not directly comparable to Ecuador's. In contrast, empirical research on child maltreatment in low- and middle-income countries in Latin America is limited (14), and studies evaluating its economic burden are almost nonexistent, despite the high prevalence of violence in the region (15). Therefore, this perspective aims to describe annualized projected governmental expenditure across three child protection modalities serving children affected by child maltreatment and other protection needs in Ecuador, and to highlight the need for a national economic burden assessment of child maltreatment through the integration of administrative data across the human development, health, and education sectors. This expenditure description should not be interpreted as a national economic-burden estimate.

## 2 Ecuador as case study

Ecuador is a middle-income country where available evidence suggests a lack of a nationally representative population-based survey that estimates the prevalence of child maltreatment, abuse, and neglect (16). In a retrospective survey conducted among university students in Quito using the International Society for the Prevention of Child Abuse and Neglect (ISPCAN) Child Abuse Screening Tool-Retrospective (ICAST-R), 69.6% reported exposure to child maltreatment (physical abuse: 47.6%; emotional maltreatment: 53.0%; sexual abuse: 15.5%) (14). These findings provide evidence of exposure to child maltreatment, while highlighting the need for nationally representative evidence in Ecuador. Available Ecuadorian economic evidence has estimated willingness-to-pay to prevent maltreatment-related deaths rather than a national cost-of-illness burden (17).

The Ministerio de Desarrollo Humano (MDH) has designed the Special Protection Services to support children experiencing situations of risk, vulnerability, or violations of their rights such as sexual abuse, abandonment, violence, parental incarceration, extreme vulnerability, and other circumstances requiring rights protection (18, 19). This system encompasses institutional care, foster care, and family-based interventions (18). Special Protection Services were previously documented under the former Ministerio de Inclusión Económica y Social (MIES) (18), now MDH, and respond to multiple rights protection needs that cannot be considered exclusively as child maltreatment.

Institutional care is an exceptional judicially ordered protective intervention, employed as a measure of last resort after the depletion of viable family preservation and community-based care alternatives in circumstances involving a child at risk and/or welfare and rights violations (20). According to the MDH, these situations may include abandonment, maltreatment, abuse, sexual exploitation, trafficking, orphanhood, having parents deprived of liberty, forced displacement, and other circumstances requiring protection. Therefore, the government is responsible for meeting all children's basic needs such as food, clothing, education, medicine, and healthcare (18). However, the available information does not permit a fully disaggregated expenditure estimate because the institutional-care cost structure includes submodalities for which beneficiary counts are not publicly reported.

Administrative records dated March 2026 were obtained from the Sistema Integrado de Información del Ministerio de Desarrollo Humano de Ecuador (SIIMDH); monthly management bulletins for Special Protection Services are publicly available on the MDH website (19). The annualized estimation was obtained from the number of users and the monthly expenditure per modality, assuming constancy over 12 months (monthly cost × 12 × number of children).

Table 1 shows the governmental expenditure for the three child protection modalities: institutional care, foster care, and family support in March 2026. Of the 1,438 users of institutional care, 239 were reported as users of direct services, and 1,199 were in services provided through agreements with external entities, which may imply different cost structures between them. Public reports listed different cost categories for trafficking-related services; however, the number of users of these services was not available. Thus, trafficking-related services were not included in the expenditure projection. On the other hand, MDH reported 18 children in foster care and 2,132 receiving family support, with estimated monthly costs per child of USD 729.93 and USD 304.86, respectively.

**Table 1:** Annualized projected governmental expenditure across special protection services in Ecuador (March 2026).

Modality	Users	Monthly cost per child (USD)	Monthly expenditure (USD)	Annualized projected expenditure (USD)
Institutional care–direct	239	1,132.70	270,715	3,248,583
Institutional care–agreement	1,199	988.40	1,185,091	14,221,099
Foster care	18	729.93	13,138	157,664
Family support	2,132	304.86	649,961	7,799,538
**Total**	**3,588**	**—**	**—**	**25,426,885**

Weighted annual expenditure per registered user: USD 7,087. *Source: SIIMDH/MDH, March 2026* *(**19**)*.

Monthly and annualized expenditure values were calculated from unrounded users × monthly cost-per-child figures and rounded to the nearest USD independently at each stage. Consequently, multiplying the rounded monthly value by 12, or summing the rounded row totals, may differ by ≤ 1–7 USD from the values shown due to accumulated rounding.

As a result, Ecuador is projected to spend approximately USD 25.4 million annually across these programmes (3,588 children), corresponding to a weighted mean of USD 7,087 per registered user. Under a deterministic sensitivity scenario allowing ±10% simultaneous variation in caseload and unit costs, the annualized projection ranges from approximately USD 20.6 million to USD 30.8 million. However, this estimation does not represent the national economic burden of child maltreatment in Ecuador. It represents an annualized estimate of public expenditure associated with the three selected Special Protection Services modalities.

This expenditure projection represents only one component of the resources allocated to child protection by the government and should not be interpreted as the economic burden of child maltreatment. It also excludes costs from children or families who never enter the formal protection system (21).

To sum up, 3,588 children were registered with MDH Special Protection Services. Institutional care, foster care, and family support are mutually exclusive; therefore, each child was counted only once in a single modality of Special Protection Services (19). This administrative denominator should not be interpreted as the population prevalence estimate of child maltreatment, abuse, or neglect in Ecuador, since evidence from population-based self-report studies and systematic reviews indicates that maltreatment identified through administrative or case-file records may represent only a subset of experiences occurring in the population (14, 21).

## 3 Limitations

This perspective has several limitations that should be considered when interpreting the expenditure projection. First, the analysis accounts only for public expenditure associated with three Special Protection Services modalities and therefore does not constitute an estimate of economic burden. Economic assessments of child maltreatment may consider a wide range of costs (3), such as the risk of developing noncommunicable diseases during adolescence and adulthood, especially those related to mental health disorders, which lead to higher rates of educational underachievement, fewer future employment opportunities, lower productivity, and more social isolation (22).

Second, the present analysis does not capture costs incurred by other sectors, such as the medical, education, judicial, and other service sectors, as well as those incurred by children or families. Third, the expenditure estimate presents an annualized projection rather than observed annual expenditure. Fourth, although the three modalities included in this analysis are mutually exclusive, they respond to multiple protection needs, including maltreatment, abandonment, parental incarceration, trafficking, extreme vulnerability, and other violations of children's rights. Therefore, the expenditure cannot be attributed exclusively to child maltreatment.

Finally, administrative protection records capture only children who enter the formal protection system, so children experiencing maltreatment who do not belong to these services are not represented in the expenditure estimate. Therefore, a national economic burden study would require population-based estimates of maltreatment.

## 4 Research agenda

Despite available public expenditure data and detailed administrative records, Ecuador has yet to produce an estimate of the economic burden of child maltreatment among children served by Special Protection Services, which is a gap that hinders evidence informed policymaking. Current expenditure projections can serve as an institutional input for a future cost-of-illness analysis, complemented by information from MDH administrative records. For instance, institutional care facilities, as caregivers and providers of basic needs (18), may track academic performance and school absenteeism as measures to estimate productivity losses (6). Furthermore, their clinical registries on children's medical status and clinical and psychological conditions provide epidemiological data required to assess healthcare resource utilization and estimate direct medical costs (6), which could strengthen the estimation (7). This administrative data remains underused in health economics and in the development of evidence-based tools for strategic decision-making. Consequently, policymaking continues to address short-term issues and provide basic services rather than understanding the long-term economic consequences and tackling the root causes of future health problems among a population that has been the victim of child maltreatment and other types of abuse or vulnerability. A coordinated prevention and systems-strengthening agenda is also consistent with international frameworks such as INSPIRE strategies from the World Health Organization for ending violence against children (23).

To date, no prior research has examined this population in Ecuador, so filling this gap requires coordinated actions among social institutions (24, 25). The Instituto Ecuatoriano de Estadística y Censos (INEC) should form an alliance with MDH to develop a national survey to determine the prevalence of child maltreatment, strengthen the data source, and improve estimates of the economic burden. Also, establishing data-sharing agreements among the MDH, the Ministerio de Salud Pública (MSP), and the Ministerio de Educación, Deporte y Cultura (MINEDEC), within their respective jurisdictions, would help overcome the information barriers that limit the development of effective public policies. In this process, academia could serve as a nexus between public institutions to transform administrative data into evidence for health economics and public policy (24). Using this compiled database would enable the estimation of the economic burden of child maltreatment, thereby reinforcing the role of research in driving evidence-based decision-making and promoting public policies to improve services for this vulnerable population.

We propose a phased research and policy agenda. First, formalize inter-institutional data-sharing agreements among the MDH, MSP, and MINEDEC. Second, link protection, health, and education registries at the individual level with ethical safeguards. Third, estimate direct medical costs and productivity indicators already collected in institutional facilities. Fourth, publish a national economic burden of child maltreatment registered under Special Protection Services. Finally, establish a robust survey that registers child maltreatment at national level to determine the prevalence of this phenomenon.
